# Oral Presentations - Abstracts of the 28^th^ Annual Congress of the SBRA. Florianópolis/SC - Brazil, 2024

**DOI:** 10.5935/1518-0557.20240062

**Published:** 2024

**O-01. Knowledge and practices in Oncofertility among Brazilian oncologists: A national survey**

**Manuela Simon Studzinski Souza^1^, Beatriz Fabiani Baixo^1^, Nicole Christine Lamin^1^, Thais Isabel Silva^1^, Marianne Ortega Ossemer^1^, Giuliano Marchetti Bedoschi^2^, Caroline Castrucci Ingold^2^**

*^1^Fundação Universidade Regional de Blumenau - Blumenau - SC - Brazil*

*^2^BedMed: Clínica de Reprodução Humana - São Paulo - SP - Brazil*

**Objective:** This study aims to understand the level of knowledge Brazilian oncologists have about fertility preservation techniques and assess their ability and willingness to recommend these options to reproductive-age cancer patients, emphasizing the importance of family planning in post-treatment quality of life.

**Methods:** We conducted a quantitative survey using a structured 18-question questionnaire distributed to 17 oncologists through an online research platform. The questions were designed to evaluate the physicians' knowledge of fertility preservation techniques, perceived barriers to implementing these techniques in their daily practice, and their comfort in discussing fertility issues with patients. The responses were analyzed using descriptive statistics and presented in charts to facilitate data visualization.

**Results:** Among the participants, 70.6% were female, and an equal proportion had less than five years of experience in oncology. It was noted that 70.6% of the physicians attended to 11-50 new cancer patients per year in the 15-39 age range. Familiarity with ASCO and ESMO guidelines was split, with 50% stating they were aware of them and 50% not, with one participant not responding. The majority of doctors (75%) were familiar with techniques such as semen cryopreservation and the use of GnRHa, but only 63% knew about ovarian tissue cryopreservation, and none were familiar with testicular tissue cryopreservation. The frequency of discussing fertility preservation varied significantly: 35.3% rarely, 35.5% occasionally, 17.6% frequently, and 11.8% always addressed the topic. Regarding referrals to human reproduction specialists, 35.3% rarely, 29.4% almost always, 17.6% always, and 11.8% sometimes made the referrals, while 5.9% never did. In this study, it became evident that the main barrier to the effective implementation of oncofertility strategies in clinical practice is the excessive cost of treatment, as indicated by 14 of the 17 participants (82.4%). Additionally, the time required to start the treatment and the lack of agility in the procedure were also highlighted issues, representing approximately 64.7% of the responses.

**Conclusion:** The results demonstrate the need to strengthen interdisciplinary collaboration between oncology and assisted reproduction, promoting greater integration of knowledge in oncofertility. The low familiarity with guidelines and significant discomfort in discussing fertility options suggest an urgent need for continuing education programs for oncologists. It is proposed to create workshops and seminars as part of ongoing professional development, as well as to integrate oncofertility as an essential component in the medical training curriculum, aiming to improve the quality of life of cancer patients by preserving the opportunity for future parenthood.

**O-02. Can alloimmune factors influence Ovarian Reserve and Polycystic Ovary Syndrome?**

**João Paolo Bilibio^1^, Arivaldo Meireles^2^, Ivan Sereno Montenegro^3^, Pablo Sales^2^, Ana Carolina Martinhago^1^, Pânila Longhi Lorenzzoni^1^**

*^1^FertiBC - Balneário Camboriú - SC - Brazil*

*^2^Pronatus - Belém - PA - Brazil*

^3^HCPA - Porto Alegre - RS - Brazil

**Objective:** Alloimmune factors are represented in particular by Natural Killer (NK) cells, which are important components of the innate immune system, are present in peripheral blood and the endometrium and have the ability to lyse target cells, in addition to producing immunoregulatory cytokines. There is evidence that a portion of repetitive pregnancy losses of unknown origin have an immunological history, one of the most investigated causes being NK cells. Furthermore, immunological factors also appear to influence ovarian function. Due to this, the objective of this study was to evaluate the possible association of alloimmune factors in ovarian reserve and polycystic ovary syndrome.

**Methods:** A cross-sectional study was carried out with women between 18 and 35 years of age with an indication for IVF due to tubal factor or male factor. All included participants had ovarian reserve assessment with measurement of FSH and estradiol, anti-Mullerian hormone and antral follicle count on the third day of the menstrual cycle.

The patients were then divided into 3 groups:

- Decreased ovarian reserve (DOR) group (antral follicle count less than 5) n=18.

- Normal ovarian reserve (NOR) group n=45 in which all ovarian reserve parameters were normal (FSH, HAM and CFA).

- Polycystic ovary syndrome (PCOS) group n=34, who met the criteria for PCOS.

All of them collected the following tests to evaluate alloimmune factors: CH50, complement C3 and NK cell activity. To carry out the “NK Cell Activity” test, effector cells (NK Cells) and target cells (K562 lineage cells) were used, after serial dilution (50:1, 25:1 and 12.5:1), incubation and use of specific dye. Target cells (K562) that were lysed were evaluated using flow cytometry.

**Results:** The mean age was similar in the DOR 33.2, NOR 33.5 and PCOS 32.2 groups, *p*=0.274. The antral follicle count in the DOR 4.3, NOR 13.7 and PCOS 24.5 groups was different in the *p*<0.001. The assessment of CH50 and C3 complement levels was similar in the 3 groups. All serial dilution assessments demonstrated increased NK cell activity in PCOS patients. NK Activity Cells 50:1: DOR 22.8, NOR 22.9, PCOS 27.8, *p=*0.017. NK Activity Cells 25:1: DOR 14.3, NOR 14.3, PCOS 17.2, *p*=0.017. NK Activity Cells 12.5:1: DOR 8.4, NOR 8.8, PCOS 10.4, *p*=0.040.

**Conclusions:** We found high levels of NK cell activity in all dilutions in patients with PCOS when compared with patients with decreased ovarian reserve (DOR) and normal reserve (NOR). We know that PCOS, due to its multiple mechanisms of action, can interfere with immunological factors, but the mechanism by which it increases the activity of NK cells and its potential role in increasing the number of follicles will require further studies following this finding.

**O-03. Association of GDF9 and BMP15 Polymorphisms and Polycystic Ovary Syndrome in patients undergoing in vitro fertilization**

**João Paolo Bilibio^1^, Arivaldo Meireles^2^, Martina Cordini^1^, Lucia Bevilacqua Borges^1^, Leticia Avi^3^, Ana Carolina Martinhago^1^**

^1^FertiBC - Balneário Camboriú - SC - Brazil.

^2^Pronatus - Belém - PA - Brazil.

^3^Univali - Itajaí - SC - Brazil.

**Objective:** Growth differentiation factor 9 (GDF9) and bone morphogenetic protein 15 (BMP15), members of the TGFβ superfamily, are potent regulators of folliculogenesis and ovulation and are both expressed in oocytes from early-stage follicles. With respect to controlled ovarian hyperstimulation phenotypes, GDF9 and BMP15 alleles have been associated with stimulation outcome. This paper aimed to assess the correlation between GDF9 and BMP15 polymorphisms, which are related to follicular development, are associate with polycystic ovary syndrome (PCOS) in patients undergoing in vitro fertilization (IVF).

**Methods:** This age-matched case-control study included two or three controls per woman undergoing controlled ovarian stimulation. Controls were women without PCOS (control group), and cases were women with PCOS group. DNA was extracted from peripheral blood and potential associations with gene polymorphisms related to follicular development GDF9 (c.398-39 C>G (intron); p.Thr149Thr (c.447C>T); p.Glu186Glu (c.546G>A); p.Val216Met(c.646G>A)) and BMP15 (c.-9C>G) were analyzed.

**Results:** Sixty-tree patients were included, 44 without PCOS and 19 with PCOS. The age (35.9 versus 32.7, P 0.001), infertility time (4.1 versus 3.8, *p*=0.705), antral follicle count (8.5 *versus* 16.3, *p*<0.001), AMH (1.0 *versus* 3.1, *p*<0.001) were found in No POS Group and POS Group, respectively. The presence of homozygosis mutant of GDF9 (c.398-39 C>G) was: Control Group 7.0% versus PCOS Group 0.0%, *p*=0.327. The presence of homozygosis mutant of GDF9 p.Thr149Thr (c.447C>T) was: Control Group 29.5% versus PCOS Group 36.8%, P 0.842. The presence of homozygosis mutant of GDF9 p.Glu186Glu (c.546G>A); was: Control Group 0.0% versus PCOS Group 5.3, *p*=0.214. There was no presence of polymorphism of GDF9 in both groups, control or PCOS. The presence of homozygosis mutant of BMP15 (c.-9C>G) was: Control Group 15.9% versus PCOS Group 0.0%, *p*=0.172.

**Conclusions:** In this study we evaluated whether polymorphisms of two members of the TGFβ superfamily, GDF9 and BMP15, could be associated with PCOS. Despite they are potent regulators of folliculogenesis and ovulation and are both expressed in oocytes from early-stage follicles, we did not find an association between these polymorphisms and PCOS, which leads us to the need to research other mechanisms in order to understand ovarian function.

**O-04. Serum Estradiol Levels and Pregnancy Outcomes in Modified Natural Cycle Euploid Embryo Transfers from Autologous Oocytes: A Retrospective Cohort Study**

**Flávia Rocha Torelli^1^, Luciana Carvalho Delamuta^1^, Cassia Raquel Teatin Juliato^2^, Georges Fassolas^1^, Luiz Fernando Oliveira Henrique^1^, João Antonio Dias Jr^1^, Carlos Roberto Izzo^1^**

^1^Clínica Originare - São Paulo - SP - Brazil.

^2^UNICAMP - Campinas - SP - Brazil.

**Objective:** To investigate the correlation between serum estradiol (E2) levels during modified natural endometrial preparation cycles for frozen embryo transfer (FET) and pregnancy outcomes.

**Methods:** This retrospective cohort study included 104 women who underwent FET in a modified natural cycle (defined as spontaneous follicular growth followed by hCG trigger administration) at a private clinic between January 2022 and December 2023. Women underwent serial ultrasound monitoring from the onset of menstruation, with assessments of endometrial thickness and dominant follicle size in the ovaries. Following the detection of the dominant follicle, serum E2 and progesterone (P4) levels were measured, and hCG subcutaneous injection was administered for ovulatory trigger. Luteal phase support comprised progesterone supplementation via vaginal or combined vaginal and oral routes, with embryo transfer occurring five days after P4 supplementation initiation. No exogenous estradiol supplementation was provided. All included cases exhibited a pre-trigger serum P4 level below 1.0 ng/mL, and only euploid blastocyst transfers from autologous oocytes on the 5th or 6th day of development were performed. Serum β-hCG testing was conducted between the 9^th^ and 10th day post-transfer, followed by ultrasound assessment four weeks post-embryo transfer in instances of positive BhCG. The study analyzed E2 levels before trigger administration and their association with pregnancy (positive β-hCG) and clinical pregnancy (ultrasound confirmation of embryo cardiac activity after six weeks of gestation). Simple and multiple logistic regression analyses (with Stepwise variable selection criteria) were utilized to explore factors influencing pregnancy and clinical pregnancy. A significance level of 5% was applied.

**Results:** The mean age at the time of FET was 37.77±2.77 years and 36.66±2.82 years at the time of *In Vitro* Fertilization (IVF). 50.96% of the women had no prior pregnancies. There were no statistically significant differences in age, body mass index (BMI), or luteal phase progesterone supplementation route between women who became pregnant and those who did not become pregnant. Ninety-eight out of 104 women underwent single embryo transfers (94.23%). The mean follicle size at hCG trigger was 16.78±1.98 mm, an average of 12.12±2.39 days to trigger. The mean endometrial thickness was 8.27±1.35 mm, with a trilaminar endometrial pattern observed in all cases. No statistically significant differences were observed between the groups for the aforementioned variables. Among women with positive β-hCG results, the mean serum β-hCG level was 270.19 ± 306.16 mIU/mL, with a statistically significant difference between those with and without clinical pregnancies (*p*<0.001). Women with E2 levels ≥ 163 ng/mL exhibited higher odds of pregnancy and clinical pregnancy, with a 2.91-fold higher likelihood of pregnancy (OR=2.91; 95% CI OR: 1.13 - 7.53; *p*=0.024) and a three-fold higher likelihood of clinical pregnancy when E2 levels exceeded 163 pg/mL (OR=3.00; 95% CI OR: 1.12-8.09; *p*=0.026). There was no statistically significant difference between the groups categorized by estradiol levels concerning pregnancies that did not progress, including biochemical pregnancies and miscarriages.

**Conclusion:** Women undergoing FET with euploid embryos from autologous oocytes in modified natural cycles demonstrate increased chances of pregnancy and clinical pregnancy when E2 levels exceed 163 pg/mL prior to hCG trigger administration. Additionally, higher β-hCG levels correlate with improved clinical pregnancy outcomes.

**O-05. Women Returning to Use Their Oocytes: a robust database evaluation of 2435 social egg freezing cycles in 10 years**

**Dóris Spinosa Chéles^1^, José Roberto Alegretti^1^, Claudia Gomes^1^, Thais Sanches Domingues^1^, Michele Panzan^1^, Eduardo Leme Alves Motta^1^, João Pedro Junqueira Caetano^2^, Mauricio Barbour Chehin^1^, Aline Rodrigues Lorenzon^1^**

^1^Huntington Medicina Reprodutiva - Eugin Group - São Paulo - SP - Brazil.

^2^Huntington Pró-Criar - Eugin Group - Belo Horizonte - MG - Brazil.

**Objective:** Due to societal changes, postponed parenthood has become a global trend. This delay can increase the risk of infertility, particularly for women over 35, where age is a known factor for meiotic errors in oocytes. Social egg freezing has emerged as a viable option for women wishing to delay motherhood, thanks to the safety and effectiveness of oocyte vitrification. Despite its growing use, follow-up studies on social egg freezing are often limited by short durations and small sample sizes. Comprehensive data on reproductive outcomes after frozen oocyte thawing (FOT) are essential for accurate counseling. This study aims to assess the rate of FOT and the reproductive outcomes (pregnancy and live birth rates) in patients who previously underwent social oocyte freezing and returned to a private ART center in Brazil to use their cryopreserved samples.

**Methods:** Retrospective cohort study including 2090 patients that underwent 2435 social egg freezing cycles between Jan/2013 and Dez/2022. Patients that underwent egg freezing for medical reasons or with the intention to perform an IVF treatment were excluded. Reproductive outcomes of patients who returned to use their oocytes during the same period were examined. Comparison between cycles that achieved an embryo transfer (ET) with those that did not and from those with ongoing pregnancy/live birth and negative pregnancy test were analyzed. Statistical analysis was conducted using Mann-Whitney, t-test, and Fisher tests, with *p*-values <0.05 considered significant.

**Results:** Mean women’s age at the time of egg freezing were 36,6±3,2 years old. There was a significant difference of mean age in 2013 (37.8±4.1) compared to 2022 (36.5±3.4, *p*<0.0001), as well as the number of oocytes frozen in 2013 (7.3±6.6) and 2022 (8.5±6.7), *p*=0.04, (cohort mean: 8.1±6.3). Number of cycles performed yearly, showed an increase rate greater than 400% between 2013 (75) to 2022 (395). Mean number of cycles per patient was 1,.2. During this period, 137 patients returned to use their oocytes (6,5%) in a mean time of 4.1±2.2 years. Ninety-five patients achieved at least one embryo transfer (69%) in 100 cycles and 42 patients hadn’t achieved an ET in 46 cycles. Mean oocyte survival post-thawing rate was 82,1%±23,6%. The main variables that showed statistical difference between cycles with an ET and no ET were: age at oocyte freeze (36.4±2.7 *vs*. 37.9±2.9, *p*=0.003); number of oocytes freeze (13.5±6.1 *vs*. 9.5±5.9, *p*=0.0003); number of blastocyst (3.3±2.3 *vs*. 1.2±1.5, *p*<0.0001), blastocyst rate per fertilized oocyte (46.7%±22.4% vs. 25.4%±28.6%, *p*<0.0001) and the proportion of euploid embryos (48.9%±24.5% *vs*. 8.5%±23.2%, *p*<0.0001). Patients that underwent PGT-A was similar between groups (48.4% *vs*. 56.5%, *p*=0.36), as well as paternal age (41.2±5.3 *vs*. 41.9±6.4, *p*=0.5) and proportion of normospermic semen samples (92.9% *vs*. 94.6%, *p*=0.9). Fourty-seven patients achieved an ongoing pregnancy/live birth (49.5%). The main variables that showed statistical difference between cycles with an ongoing pregnancy/live birth and negative pregnancy outcome after an ET were: number of blastocyst (3.9±2.6 *vs*. 2.8±1.9, *p*=0.02), blastocyst rate per fertilized oocyte (53.0%±22.1% *vs*. 41.2%±21.4%, *p*=0.005) and the proportion of cycles with PGT-A (59.6% *vs*. 37.3%, *p*=0.04).

**Conclusions:** Although the return rate of the 2090 patients was only 6.5%, we can expect this rate will increase in the coming years, once the number of egg freezing cycles has increased more than 400% over the last 10 years. Half of the patients achieved a pregnancy with a mean age of 36,6 years old at the time of egg freezing. Perhaps, whether they had performed oocyte freezing younger, this outcome could be better. Growing awareness campaigns have emphasized the importance of undertaking egg freezing as early as possible and should address the limitations of ART procedures in older women.

**O-06. Are day-6 euploid blastocyst morphokinetic patterns as reliable for predicting clinical pregnancy as day-5 euploid blastocysts?**

**Beatriz Aiello^1^, Bruna Lourenço^1^, Mariana Nicolielo Barreto^1^, Dóris Spinosa Chéles^1^, Mauricio Barbour Chehin^1^, José Roberto Alegretti^1^, Aline Rodrigues Lorenzon^1^**

^1^Huntington Medicina Reprodutiva - Eugin Group - São Paulo - SP - Brazil.

**Objective:** Morphokinetics has become an essential tool in embryo selection for in vitro fertilization (IVF). Time-lapse imaging technology allows for continuous monitoring of embryos, providing detailed information on developmental milestones. It has been shown that morphokinetic parameters are correlated with clinical outcomes such as pregnancy rates. Most commercial software uses morphokinetic parameters to choose and rank the best embryo for transfer. It is important to highlight that these software programs were designed with data from day 5 blastocysts (D5). The association of morphokinetic parameters with reproductive outcomes for day 6 blastocysts (D6) is still unknown. Here, we evaluated the main morphokinetic parameters in euploid D5 and D6 blastocysts that reached a clinical pregnancy compared to those with a negative outcome.

**Methods:** This cohort study included morphokinetic data from 409 biopsied embryos from 364 patients (mean of 1.1 euploid embryos/patient) undergoing autologous IVF with preimplantation genetic testing for aneuploidies (PGT-A) between 2018-2023. All embryos were cultured in a time-lapse system (Embryoscope Plus, Vitrolife). Blastocysts’ inner cell mass (ICM) and trophoectoderm (TE) were morphologically graded according to Gardner’s system. The morphokinetic parameters considered for analysis were: time of pronucleus fading (tPNf), time to 2-cell (t2), time to 3-cell (t3), time to 4-cell (t4), time to 5-cell (t5), time to 8-cell (t8), and time to blastulation (tB). Mann-Whitney, t-test, and Fisher's test were used as appropriate. A *p*-value of <0.05 was considered significant.

**Results:** The mean maternal age was 39.0±3.5 years. All morphokinetic parameters analyzed were significantly different when comparing D5 and D6 embryos: tPNf: 23.4±3.0 *vs*. 25.0±3.2; t2: 26.5±3.5 *vs*. 27.6±3.3; t3: 37.0±4.1 *vs*. 38.7±4.4; t4: 38.6±4.7 *vs*. 41.0±5.0; t5: 49.7±6.8 *vs*. 52.9±7.1; t8: 59.3±10.0 vs. 63.5±10.6; tB: 107.2±10.2 *vs*. 119.9±8.6, all with *p*<0.0001. Maternal age was similar between D5 and D6 embryos (38.9±3.6 *vs*. 39.1±3.4, *p*=0.6). A higher proportion of grade A compared to B+C for ICM and TE were observed in D5 embryos (ICM grade A: 50% *vs*. 30.8%, *p*=0.0003; TE grade A: 36.6% *vs*. 23.6%, *p*=0.004 for D5 *vs*. D6, respectively). Surprisingly, clinical pregnancy rates were not different between D5 and D6 euploid embryos (46.7% *vs*. 44.3%, *p*=0.6). Morphokinetic patterns between embryos that achieved a clinical pregnancy and those with a negative result were different in D5 blastocysts at t8 and tB (57.1±7.9 *vs*. 61.1±11.3, *p*=0.004 and 105.8±9.3 *vs*. 108.4±10.9, *p*=0.03, respectively). A higher proportion of grade A ICM and TE grades were observed in D5 embryos that achieved a clinical pregnancy (ICM: 58.1% *vs*. 42.9%, *p*=0.01; TE: 45.7% *vs*. 28.6%, *p*=0.004, compared to negative outcomes). Interestingly, in D6 blastocysts, none of the morphokinetic parameters were different between embryos resulting in clinical pregnancy and those with negative outcomes. However, the morphology grades of ICM and TE were different between these groups: ICM grade A: 40.5% *vs*. 23.7%, *p*=0.02; TE grade A: 33.8% *vs*. 18.3%, *p*=0.03 (clinical pregnancy *vs*. negative outcome, respectively). Maternal age was similar between clinical pregnancy and negative outcome groups in both D5 and D6 embryos (D5: 38.7±3.0 *vs*. 39.2±4.0, *p*=0.22; D6: 38.9±3.8 *vs*. 39.2±3.7, *p*=0.6).

**Conclusions:** Our study shows that morphokinetic parameters are effective predictors of clinical pregnancy in euploid day 5 (D5) blastocysts, with successful embryos developing earlier, particularly in time to blastulation (tB). In contrast, day 6 (D6) blastocysts did not show significant differences in morphokinetic parameters between those resulting in clinical pregnancy and those with negative outcomes. However, ICM and TE morphology remain reliable for selecting D6 embryos with high implantation potential. These findings suggest caution in using artificial intelligence-based scoring systems designed for D5 blastocysts on D6 embryos. Further research is needed to improve selection criteria and ART outcomes for D6 blastocysts.

**O-07. Does the ejaculatory abstinence time influence on semen quality? A case-control study in a private IVF center**

**Taynara Priscilla de Freitas^1^, Fabricio Santana dos Santos^1^, Lais Maroni Portugal^1^, Martina Caroline Stapenhorst^2^, Ana Carolina Cardillo Fernandes^2^, Thiago Augusto Campos Luders Lofti^2^, Leticia Kaory Tamashiro^2^, Ana Luisa Menezes Campos^1^, Andrea Belo^2^, José Roberto Alegretti^2^, Dóris Spinosa Chéles^2^, Aline Rodrigues Lorenzon^2^**

^1^Huntington Pró-Criar - Eugin Group - Belo Horizonte - MG - Brazil.

^2^Huntington Medicina Reprodutiva - Eugin Group - São Paulo - SP - Brazil.

**Objective:** Couple infertility is a major challenge in assisted reproductive technology (ART) treatments, with male factor accounting for approximately 50% of infertility cases. Semen evaluation is essential for identifying male factor infertility, and the sperm parameters analyzed can be affected by the length of ejaculatory abstinence. The World Health Organization (WHO) Laboratory Manual for the Examination and Processing of Human Semen recommends an abstinence period of 2-7 days before semen evaluation, while the European Society of Human Reproduction and Embryology (ESHRE) recommends a period of 3-4 days. However, it is unclear if these guidelines are effective for infertile men undergoing ART. Shortening or prolonging ejaculatory abstinence may indicate ways to improve semen quality. The objective of this study was to analyze different periods of ejaculatory abstinence, their influence on semen quality, and the associated reproductive outcomes across different cycles in the same couple.

**Methods:** This is a case-control cohort study involving couples undergoing IVF where at least two cycles with different periods of ejaculatory abstinence were performed. The variables considered for analysis included the time of abstinence (days), maternal and paternal ages, sperm concentration, motility, morphology according to Kruger’s criteria, number of oocytes retrieved (OPU), number of mature oocytes (MII), percentage MII, number of fertilized oocytes, fertilization rate, number of blastocysts formed, percentage of blastocysts per fertilized embryo, and clinical pregnancy rate. Statistical analysis was performed using the Wilcoxon matched-pairs test and Fisher's test, with a p-value of less than 0.05 considered significant.

**Results:** A total of 201 couples and 402 cycles were analysed. The mean maternal age was 38.5±3.5 years and the mean paternal age was 39.5±3.5 years. The mean time of abstinence was 2.5±1.1 days for the shortest cycle and 4.2±2.5 days for the longest cycle (*p*<0.001). Sperm concentration and motility were higher in cycles with the longest abstinence compared to the shortest (80.9±61.8 *vs*. 70.9±56.3, *p*=0.004 and 50.2±47.2 *vs*. 44.1±41.4, *p*=0.01). Semen morphology parameters were also similar between the longest and shortest abstinence cycles (2.2±1.3 *vs*. 2.3±1.5, *p*=0.40). The proportion of normospermic semen analyses was similar between the groups (15.4% *vs*. 17.9%, *p*=0.60). No differences were observed in the number of oocytes retrieved (7.1±4.9 *vs*. 7.2±5.3, *p*=0.6), mature oocytes (5.2±4.0 *vs*. 5.4±4.0, *p*=0.22), percentage of mature oocytes (72.0%±26.0% *vs*. 75.8%±23.8%, *p*=0.08), number of fertilized oocytes (4.0±3.2 *vs*. 4.2±3.3, *p*=0.21), fertilization rate (71.3%±30.1% *vs*. 76.1%±27.8%, *p*=0.14), number of blastocysts formed (1.9±1.7 *vs* 1.9±1.7, *p*=0.57), and percentage of blastocyst formation (44.9%±34.6% *vs*. 46.0%±33.0%, *p*=0.58) between the longest and shortest cycles. Clinical pregnancy rates were also similar (37.5% *vs*. 37%, *p*=0.9).

**Conclusions:** This study reveals that varying the length of ejaculatory abstinence influences certain semen parameters, such as sperm concentration and motility, which were higher in cycles with longer abstinence periods. However, other parameters, including sperm morphology, fertilization rates, blastocyst formation, and clinical pregnancy rates, were not significantly affected by the length of abstinence. These findings suggest that while adjusting the abstinence period may improve specific sperm characteristics, it does not necessarily translate to improved reproductive outcomes in ART cycles. Therefore, individualized recommendations on ejaculatory abstinence should be considered. Further research, such as the impact of abstinence period on sperm DNA fragmentation, is warranted to explore the optimal abstinence period for enhancing ART outcomes.

**O-08. Does immature retrieved oocyte number have an impact on laboratorial outcomes?**

**Vanessa Devens Trindade^1^, Marta Ribeiro Hentschke^1^, Fabiana Mariani Wingert^1^, Isadora Badalotti-Teloken^1^, Natália Fontoura Vasconcelos^1^, Victória Campos Dornelles^1^, Adriana Cristine Arent^1^, Carolina Comissoli Fernandes^1^, Alvaro Petracco^1^, Mariangela Badalotti^1^**

^1^Fertilitat Centro de Medicina Reprodutiva - Porto Alegre - RS - Brazil.

**Objective:** Studies have suggested higher rates of metaphase II oocytes (MII) are associated with higher live birth rates after *in Vitro* Fertilization (IVF). According to the Vienna Consensus, the oocyte maturity rate (OMR) is among the most important parameters for oocyte quality prediction, but other possible parameters are still under study. Thus, the aim of this study is to evaluate if a higher proportion of immature oocytes in the retrieved cohort has a negative impact on blastulation rates after IVF.

**Methods:** Retrospective, observational study performed at a reproductive medicine center. A total of 2542 IVF cycles between 2019 and 2023 were included. Cycles with different sperm source from autologous ejaculate or donor and ≥ 42 years old female age were excluded. The sample was divided into two groups according to the OMR: G1 ≥ 75% (n=1485) and G2 <75% (n=1057). Variables analyzed were: female and male age, total number of oocytes retrieved, number of MII, fertilization rate and blastulation rate. Variables were compared between groups and expressed as mean±SD and n (%). ANOVA and chi square tests were applied (considering *p*<0.05 statistically significant).

**Results:** When comparing groups G1 and G2, the following results were found, respectively: mean female age, yo (36.56±3.83 *vs*. 36.84±3.76 *p*=0.066), mean male age, yo (38.89±5.57 *vs*. 36.84±3.76, *p*=0.877), mean oocyte number (10.67±7.79 *vs*. 11.53±8.38, *p*=0.008), mean mature oocyte number (9.22±6.53 *vs*. 6.79±5.52), fertilization rate, % (76.9 *vs*. 75.7, *p*=0.063), blastulation rate, % (53.0 *vs*. 46.7, *p*<0.001).

**Conclusion:** The results showed that regardless of the total number of oocytes collected in the groups, the fertilization rate did not have a significant difference. Nevertheless, a higher percentage of immature oocytes is associated with lower blastulation rates after IVF, suggesting that a higher percentage of immature oocytes may also be correlated to worse mature oocyte quality and competence, leading to worse IVF laboratory results.

**O-09. Correlation of oocyte quality assessment by artificial intelligence to blastocyst development, and ovarian stimulation protocols**

**Daniela Paes Almeida Ferreira Braga^1^, Amanda Souza Setti^1^, Maite Del Collado^2^, Jullin Fjeldstad^3^, Natalie Mercuri^3^, Parisa Mojiri^3^, Maria Cecília Erthal^4^, Maria Cecília Maria Cecília Cardoso^4^, Edson Borges Jr.^5^**

*^1^Fertility Medical Group, and Sapientiae Institute - São Paulo - SP - Brazil*

*^2^Science Creating Lives - São Paulo - SP - Brazil*

*^3^Future Fertility - Canada*

*^4^Vida Centro de Fertilidade/FERTGROUP Medicina Reprodutiva - Rio de Janeiro - RJ - Brazil*

^5^Fertility Medical Group/FERTGROUP Medicina Reprodutiva and Sapientiae Institute - São Paulo - SP - Brazil

**Objective:** MAGENTA™ is an artificial intelligence (AI) tool that assesses images of mature denuded oocytes to provide an analysis shown to significantly correlate with subsequent blastocyst development and quality, consistently outperforming embryologists in this task. MAGENTA™ reports provide an objective measure of oocyte quality, including images and individualized scores (0-10) that predict blastocyst formation for each oocyte. Therefore, this AI tool can be extremely useful not only for predicting embryo implantation potential but also for improving embryo selection for transfer, facilitating the management of patient expectations, and allowing the transfer of fewer embryos, thereby reducing multiple pregnancies and associated risks. Furthermore, it can help us better understand the biology of the oocyte and its response to different protocols of controlled ovarian stimulation, and pituitary blockage. Therefore, the objectives for this study were to investigate: (i) whether MAGENTA™ oocyte score (MS) may predict blastocyst development and morphokinetic factors, (ii) the influence of pituitary blockage on oocyte quality, assessed by MAGENTA™ and, (iii) to evaluate the influence of the controlled ovarian stimulation (COS) protocol on oocyte quality, assessed by MAGENTA™.

**Methods:** For this study, MS was applied to 7,783 oocyte images generated by the time-lapse imaging system (TLI). For the primary objective, oocytes were split into two groups according to the blastocyst development outcome: Blastocyst (n=4,612) and Non-blastocyst Group (n=3,171) and embryo morphokinetic data was analyzed. For the secondary objective, oocytes were split into groups depending on the protocol used to prevent the LH surge: progestin-primed (n=707) and GnRH-antagonist group (n=5,537) and, for the third objective, (iii) oocytes were divided into groups based on the COS protocol: FSH-only (n=2,270) or FSH + LH (n=2,879). The correlation of MS to blastocyst formation and morphokinetic data: timing to pronuclei appearance and fading (tPNa and tPNf), two (t2), three (t3), four (t4), five (t5), six (t6), seven (t7), and eight cells (t8), morulae (tM), start of blastulation (tSB) and blastulation (tB), was evaluated. In a second analysis, the correlation of the pituitary blockage and COS protocol to MS were evaluated.

**Results:** The MS was significantly higher in oocytes that reached the blastocyst stage when compared to those that did not achieve the blastocyst stage (6.5 *vs*. 5.5, *p*<0.001 Welch’s t-test). A negative correlation between MS and morphokinetic factors was noted (indicating shorter durations between developmental stages is favorable), especially for early embryos divisions (tPNa, tPNf, t2, t4, t6, t7, and t8). No differences were observed on MS when different protocols were used to prevent the LH surge (*p*=0.191 Welch’s t-test). When COS protocols were compared, no significant differences were noted when the whole group was evaluated (*p*=0.225), but when the oocytes were divided by age (<35 and ≥35), a higher MS was observed in oocytes derived from cycles stimulated with FSH + LH (5.9) when compared with dose stimulated exclusively with FSH for the ≥35 age group (5.6, *p*<0.01 Welch’s t-test).

**Conclusions:** In this study, an AI model for predicting blastocyst formation was validated. The correlation between the MS and morphokinetic parameters adds a layer of confidence to the model. It's reassuring since the connection between morphokinetic data, and the embryo implantation potential is already known. Additionally, our findings suggest that older patients might benefit at the oocyte level from the addition of LH in the COS. This corroborates with previous studies indicating that embryos from cycles stimulated with LH tend to develop faster and have an increased chance of implantation, especially for women of advanced age.

**O-10. The effect of embryo mosaicism on morphokinetic events and KIDScore in Preimplantation Genetic Testing: A retrospective cohort study**

**Amanda Souza Setti^1^, Daniela Paes Almeida Ferreira Braga^1^, Maite Del Collado^2^, Patricia Guilherme^3^, Renato Tomioka^4^, Vinicius Medina Lopes^5^, Assumpto Iaconelli Jr.^6^, Edson Borges Jr.^6^**

^1^Fertility Medical Group, and Sapientiae Institute - São Paulo - SP - Brazil.

^2^Science Creating Lives - São Paulo - SP - Brazil.

^3^Fertility Medical Group / FERTGROUP Medicina Reprodutiva - Sao Paulo - SP - Brazil.

^4^Vida Bem Vinda / FERTGROUP Medicina Reprodutiva - São Paulo - SP - Brazil.

^5^Instituto Verhum / FERTGROUP Medicina Reprodutiva - Brasília - DF - Brazil.

^6^Fertility Medical Group / FERTGROUP Medicina Reprodutiva, and Sapientiae Institute - São Paulo - SP - Brazil.

**Objective:** Over the last decades, efforts have been made to assess the genetic status of preimplantation embryos, distinguishing chromosomally normal embryos, considered to have high developmental potential, from their aneuploid siblings. Therefore, the preimplantation genetic testing for aneuploidy (PGT-A) emerged as an approach to analyse embryos’ DNA for determining genetic abnormalities. Previous studies have demonstrated that embryo development is affected by intrinsic embryonic machinery; therefore, it could be hypothesized that the embryo chromosomic status could affect embryo development. We hypothesized that specific morphokinetics events may be correlated with embryos mosaicism, therefore the identification of these events may be a powerful toll in assisted reproduction, improving the selection of the embryo with the highest potential for implantation, without the detrimental effects of embryo biopsy. Thus, the aim of this study was to investigate the effect of low and high embryo mosaicism on embryo morphokinetics events in a time-lapse imaging (TLI) culture system.

**Methods:** This retrospective cohort study was performed between 2020-January and December-2023. Kinetic data were analyzed in 1189 embryos, derived from 365 patients undergoing ICSI cycles with PGT-A, individually cultured in a TLI incubator until day five. The determination of euploidy, aneuploidy, and mosaicism was performed by using next-generation sequencing (NGS) at a single reference laboratory. Embryos with chromosomal aneuploidy < 50% were considered low-mosaics, and those between 50 and 70% were considered high-mosaics. Generalized linear mixed models, controlled for potential confounders, followed by Bonferroni post-hoc, were used to compare the morphokinetic variables and known implantation data score (KIDScore)-day 5 amongst euploid (n=338) aneuploid (n=566), low-mosaic (n=186) and high-mosaic embryos (n=99). Recorded morphokinetic variables were: (i) Timing to pronuclei appearance (tPNa) and fading (tPNf), timing to two (t2), three (t3) cells, four (t4), five (t5), six (t6), seven (t7), eight (t8) cells, morulation (tM), and blastulation (tB). (ii) Durations of the second (cc2) and third (cc3) cell cycles, timing to complete t2-tPNf (s1), t4-t3 (s2), and t8-t5 (s3) synchronous divisions. (iii) The incidences of multinucleation at the 2-cell or 4-cell stages. Given multiple cycles were present per couple and multiple embryos per couple, two random effects were added to account for the correlation between embryos of different cycles of the same couple, and embryos within the same cycle.

**Results:** When compared to euploid embryos, aneuploid embryos showed significantly longer tPNa, tPnf, t2, t3, t4, t6, t7, t8, tM, tsB, and tB. Additionally, aneuploid embryos presented longer times to complete s1, s2, and s3. Embryos classified as high-mosaic also exhibited a slower division pattern compared to euploids for the following parameters: t5, t6, t7, t8, tM, tsB, tB, s1, s2, and s3. Low-mosaic embryos were slower than euploids only in the later stages of development, such as tM and tB. The KIDScore ranked significantly differently among euploid, low-mosaic, high-mosaic, and aneuploid embryos, with euploid embryos showing a significantly higher score compared to low-mosaic embryos, while high-mosaic and aneuploid embryos presented lower scores (6.7±0.12, 5.7±0.16, 5.6±0.09, 5.3±0.24, *p*<0.001). No significant differences were noted in the durations of cc2 and cc3, nor in the incidences of multinucleation.

**Conclusions:** High-mosaic embryos display a developmental pattern resembling that seen in aneuploids, characterized by slower divisions from the 5-cell stage onwards, while low-mosaic embryos show more pronounced developmental delays in later stages (tM and tB). The KIDScore effectively distinguishes among aneuploid, euploid, and mosaic embryos, successfully differentiating between low-mosaic and high-mosaic types. These findings suggest that, similar to aneuploid embryos, high-mosaic embryos exhibit more prominent developmental deficiencies than low-mosaic and euploidy embryos, underscoring the importance of careful consideration when deciding on the transfer of such embryos.

**O-11. Letrozole ovulation induction plus hCG trigger versus hormonal replacement therapy for single euploid blastocyst transfer**

**Matheus Roque^1^, Gustavo Nardini Cecchino^1^**

^1^Mater Prime & Mater Lab - São Paulo - SP - Brazil.

**Objective:** To evaluate if letrozole ovulation induction associated to hCG trigger may improve the clinical outcomes in frozen-thawed single euploid blastocyst transfer in women with regular menstrual cycles when comparing to hormonal replacement therapy (HRT)

**Methods:** A retrospective cohort study was conducted between April 2022 and September 2023. Patients with regular menstrual cycles undergoing single embryo transfer of euploid day five or day six blastocysts were included in the study. During the study period, 479 FET cycles of women with regular menstrual cycles aged < 45 years performing single euploid blastocyst transfer were evaluated. Patients were divided into the HRT group (n=316) and the letrozole group (n=163). In the HRT group, the endometrial priming was performed with estradiol valerate and the luteal phase support starting five days before the FET with vaginal micronized progesterone plus oral dydrogesterone. In the Letrozole group, the patients used Letrozole 5mg daily on cycle days 3-7. When the endometrial thickness reached ≥7mm and at least one follicle ≥14mm, an hCG trigger was performed, and the FET was programmed for hCG+7 days. The primary outcome was the ongoing pregnancy rate (OPR).

**Results:** A total of 479 FET cycles were included for analysis (316 HRT cycles and 163 letrozole induction cycles). When comparing the two groups, there was no statistical difference in terms of maternal age, BMI, ovarian reserve tests, protocols for controlled ovarian stimulation, and endometrial thickness before programming the FET. The clinical outcomes were significantly higher in the letrozole induction FET group than in the HRT group. After adjusting for confounding factors, pregnancy rates (PR), clinical pregnancy rates (CPR), and OPR was consistently higher in the letrozole group. The PR was 79% in the letrozole group versus 67% in the HRT group, with a relative risk (RR) in favor of Letrozole of 1.17 (95% CI, 1.05-1.31; *p*=0.004). The CPR was 74% in the letrozole group versus 64% in the HRT group, with a RR in favor of Letrozole of 1.16 (95% CI, 1.03-1.31; *p*=0.017). The OPR was 68% in the letrozole group versus 59% in the HRT group, with a relative risk in favor of Letrozole of 1.15 (95% CI, 1.01-1.33; *p*=-0.04).

**Conclusion:** Letrozole ovulation induction plus hCG trigger improves the clinical outcomes of frozen-thawed euploid blastocyst transfer compared to hormonal replacement therapy (HRT) in women with regular menstrual cycles.

**O-12. Evaluation of Nail Polish Embryotoxicity Using the Mouse Embryo Assay (MEA)**

**Izabella da Silva Duarte^1^, Michelle Guimarães dos Santos^1^, Caroline dos Santos da Fonseca^1^, Mariana Santos Costa^1^, Luanna Prudencio de Araujo^1^, Marcel Frajblat^1^**

^1^Universidade Federal do Rio de Janeiro - Rio de Janeiro - RJ - Brazil.

**Objective:** To evaluate the embryotoxicity of two types of nail polishes using the Mouse Embryo Assay (MEA). The United States Food and Drug Administration (FDA) requires the MEA test to validate a product as non-embryotoxic, which must achieve a blastocyst formation rate of over 80%.

**Methods:** Sexually mature female mice (6-8 weeks) were subjected to superovulation to increase the number of available embryos by intraperitoneal injection of 5 IU of equine chorionic gonadotropin (eCG). After 48 hours, an injection of 5 IU of human chorionic gonadotropin (hCG) was administered to induce ovulation. Two days after mating, two-cell embryos were collected and divided into groups. Two types of nail polish were tested: a white conventional formulation nail polish from the brand Drica and a red gel nail polish from the brand Risqué, applied to the base of the dish near the medium drop or on the inner surface of the lid. The two-cell embryos were divided into five groups: 1) Conv NP lid - conventional nail polish applied to the lid; 2) Conv NP base - conventional nail polish applied to the base; 3) Gel NP lid - gel nail polish applied to the lid; 4) Gel NP base - gel nail polish applied to the base; and 5) Control. Embryos were cultured in 50µl drops of HTF medium (Global^®^ Total^®^) in an incubator with 5% CO_2_ and high humidity. The blastocyst formation rate was analyzed over a 72-hour period. Results were analyzed using the chi-square test. The project was approved by the Animal Ethics Committee of UFRJ (protocol #169/23).

**Results:** A total of 298 two-cell embryos were divided among the groups. The conventional nail polish showed a toxic effect when applied to the base of the dish, with no embryonic development (0% blastocyst rate) in this group. When the conventional nail polish was applied to the lid, there was a reduced blastocyst rate (60.8%) compared to the control group (85.7%). The gel nail polish did not show any embryotoxic effect, with blastocyst rates above 80% when applied to the base of the dish (88.7%) and to the lid (92.7%).

**Conclusion:** The study suggests that conventional nail polish, formulated with formaldehyde, ethyl acetate, and nitrocellulose, presents embryotoxic effects. No embryotoxicity was observed with the gel nail polish. Concerns about the embryotoxicity of cosmetics should be ongoing but based on scientific observations. The MEA is currently the primary test for determining the embryotoxicity of materials used in assisted reproduction.

